# The effects of graded levels of calorie restriction: III. Impact of short term calorie and protein restriction on mean daily body temperature and torpor use in the C57BL/6 mouse

**DOI:** 10.18632/oncotarget.4506

**Published:** 2015-07-22

**Authors:** Sharon E. Mitchell, Camille Delville, Penelope Konstantopedos, Davina Derous, Cara L. Green, Luonan Chen, Jing-Dong J. Han, Yingchun Wang, Daniel E.L. Promislow, Alex Douglas, David Lusseau, John R. Speakman

**Affiliations:** ^1^ Institute of Biological and Environmental Sciences, University of Aberdeen, Aberdeen, Scotland, UK; ^2^ Key laboratory of Systems Biology, Innovation Center for Cell Signaling Network, Institute of Biochemistry and Cell Biology, Shanghai Institute of Biological Sciences, Chinese Academy of Sciences, Shanghai, China; ^3^ Chinese Academy of Sciences Key Laboratory of Computational Biology, Chinese Academy of Sciences-Max Planck Partner Institute for Computational Biology, Shanghai Institutes for Biological Sciences, Chinese Academy of Sciences, Shanghai, China; ^4^ State Key Laboratory of Molecular Developmental Biology, Institute of Genetics and Developmental Biology, Chinese Academy of Sciences, Chaoyang, Beijing, China; ^5^ Department of Pathology, University of Washington at Seattle, Seattle, Washington, USA

**Keywords:** Gerotarget, dietary restriction, protein restriction, calorie restriction, body temperature, torpor

## Abstract

A commonly observed response in mammals to calorie restriction (CR) is reduced body temperature (T_b_). We explored how the T_b_ of male C57BL/6 mice responded to graded CR (10 to 40%), compared to the response to equivalent levels of protein restriction (PR) over 3 months. Under CR there was a dynamic change in daily T_b_ over the first 30–35 days, which stabilized thereafter until day 70 after which a further decline was noted. The time to reach stability was dependent on restriction level. Body mass negatively correlated with T_b_ under *ad libitum* feeding and positively correlated under CR. The average T_b_ over the last 20 days was significantly related to the levels of body fat, structural tissue, leptin and insulin-like growth factor-1. Some mice, particularly those under higher levels of CR, showed periods of daily torpor later in the restriction period. None of the changes in T_b_ under CR were recapitulated by equivalent levels of PR. We conclude that changes in T_b_ under CR are a response only to the shortfall in calorie intake. The linear relationship between average T_b_ and the level of restriction supports the idea that T_b_ changes are an integral aspect of the lifespan effect.

## INTRODUCTION

Calorie restriction (CR) (sometimes called dietary restriction or DR) [1, 2] is one of the few known environmental modulations that leads to an increase in animal lifespan [3]. CR has been shown to be effective in a wide range of species [4–6] although its impact is not universal [7]. Given the contrasting results between two major studies of CR on non-human primates there has been much recent interest in the potentially important roles of different macronutrients within the diet [6, 8, 9] and renewed interest in the potential importance of background genotype on the magnitude and direction of the effect [10–15]. However, despite a concentrated effort for several decades the underlying molecular mechanisms by which CR exerts its life enhancing effects remain unclear [16–21].

A commonly observed response to CR among endothermic species is a reduction in body temperature, particularly in rodents [4, 22–28] but also including non-human primates [29–31]. This effect has also been observed among human subjects under voluntary restriction [32] or during randomized controlled CR trials (CALERIE) [33, 34]. Transgenic manipulation of mice to elevate levels of uncoupling protein 2 (UCP2) in hypocretin expressing neurons in the hypothalamus leads to a regional increase in brain temperature adjacent to the temperature sensing mechanism in the pre-optic area, which precipitates a compensatory decline in peripheral body temperature by about 0.5–0.6^°^C and a 12–20% extension of lifespan [35]. Consequently, the body temperature response to CR may be a critical component of the lifespan extension effect. Koizumi et al (1996) suggested that as much as 50% of the life extending effects of CR might be due to body temperature lowering [36]. Supporting this view the Ames and Snell dwarf mice, which are both long lived, have lower body temperatures than wild type controls [37]. Lowered body temperature may also be an important factor influencing the lifespan of ectothermic animals [38, 39] although in this case the mechanisms may be different from those at play in endotherms. Of particular relevance is the fact that temperature mediated impacts on ectotherms appear independent of CR mediated extensions in lifespan [40], with CR delaying the onset, and temperature altering the gradient, of the mortality trajectory [27].

Because graded levels of restriction lead to a graded response in lifespan [41, 42] the factors that relate to graded levels of restriction are of particular interest. We have shown previously that graded levels of CR lead to reductions in body mass (BM) and altered body composition over a period of about 30 days following the initiation of restriction [43]. This reduction involves a preferential utilization of both body fat and structural tissue, with more minor impacts on the reproductive and vital organs [43]. These changes in body composition lead to parallel changes in various hormonal factors, notably leptin, insulin and insulin like growth factor 1 (IGF-1) [44]. In the current paper we sought to explore whether changes in body temperature under graded levels of restriction are coordinated with or disconnected from these alterations in body composition and hormonal changes. Previous work has shown that late onset (14 month old mice) 27% CR has complex effects on the minute by minute changes in body temperature, physical activity patterns and circadian rhythms [45]. We address here only the effects of CR at the scale of the mean body temperature averaged over entire days. We have addressed the impact of graded restriction on these microscale changes in circadian temperature and physical activity responses in a separate paper (Lusseau *et al*, submitted) [44, 46].

Although it was suggested in the 1970s that the key impact of DR was the shortfall in calorie intake (hence why it is generally called CR rather than DR [2]) more recently it has been suggested that the responses to reducing the availability of food depend less on the shortfall in calories, and more on the shortfall of specific macronutrients—notably protein [47]. We have shown that the changes in body composition under graded levels of CR are not recapitulated by the same levels of protein restriction (PR) [43]. In the current study we sought therefore to also explore whether the responses of body temperature to graded levels of CR (where both calorie and protein supply change in tandem) were similar to the responses to equivalent restrictions in the levels of dietary protein (PR), where profound changes in body composition are not observed.

## RESULTS

### Calorie restriction (CR)

#### Mean daily body temperature

**Figure 1:**
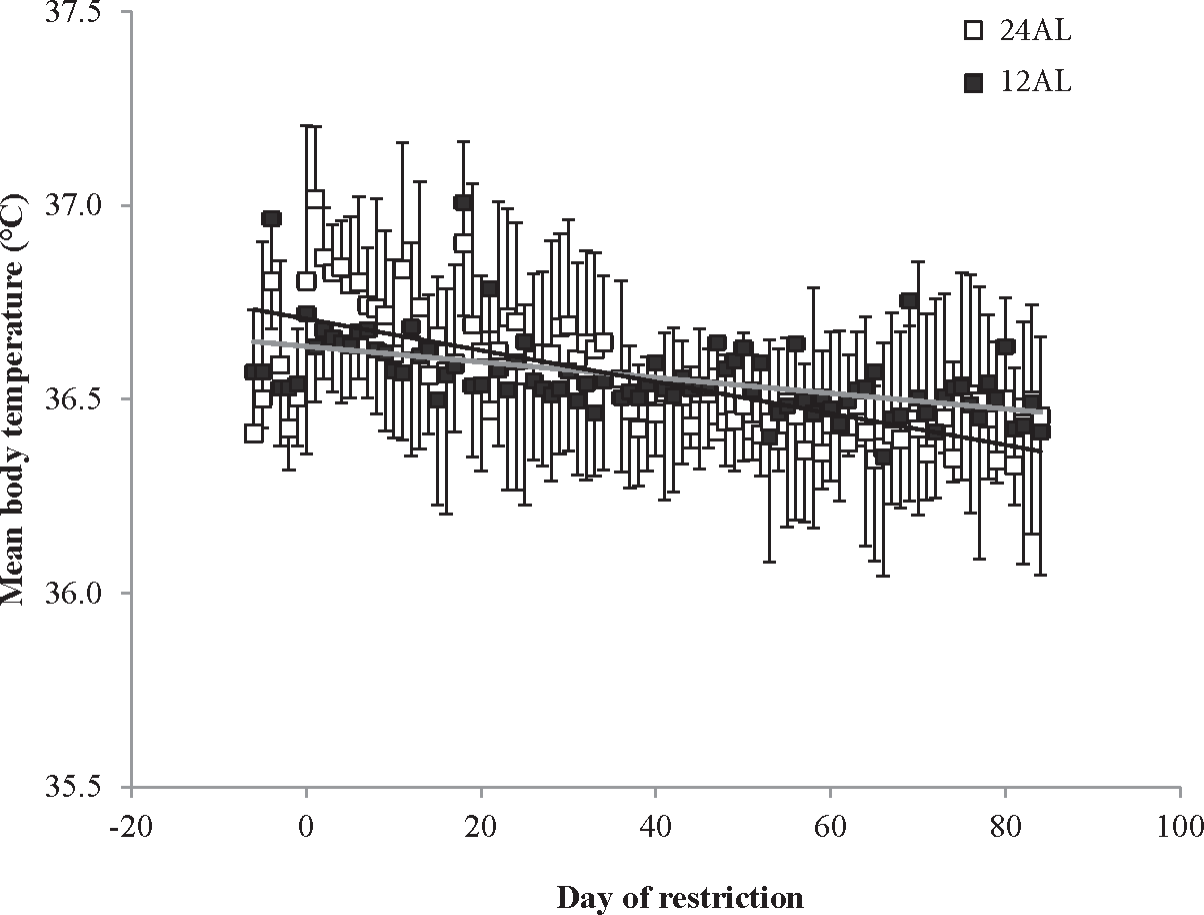
Mean daily body temperatures of the two *ad libitum* (AL) fed groups throughout the experiment The animals with 24 hour AL food access (24AL) are shown as open symbols and the mice with 12 hour AL food access (12AL) are shown as closed symbols. The x-axis is the day of treatment with Day is the start of ‘calorie restriction’. Prior to that (negative days) all mice were at baseline and fed only during the 12 hrs of darkness. (*n* = 7 in the 24AL and 8 in the 12AL groups). The grey line shows fitted regression over days 0 to 83 for 24AL animals and the black line fitted regression for 12AL animals over the same period. Results are expressed as mean ± SD.

All mice, which were exposed to the same 12AL (food provided *ad libitum* over the 12 hr of darkness) feeding regime during baseline, quickly acclimated to only having their food available during darkness and consumed their daily energy requirements within the restricted time that it was accessible. The mean daily body temperature over the baseline period across all the mice was 36.6 ± 0.3^°^C (SD, *n* = 45 individuals measured over 7 days). No significant difference between the six groups was found over this period (GLM, F_(5, 269)_ = 0.45, *p* = 0.813). On the first day of the manipulation the mean daily body temperature of the 24AL mice (food available 24 hr *ad libitum*) increased by 0.4^°^C to an average of 37.0 ± 0.2^°^C (Figure 1). This was significantly higher than the 12AL animals which averaged 36.6^°^C on the same day (one-way ANOVA: F_(1, 14)_ = 20.58, *p* < 0.0001) and the level remained significantly elevated for a period of 8 days (ANOVA on mean over the 8 days *p* < 0.05). This period of elevation corresponded to a period of hyperphagia and elevated BM in the 24AL group following the transition from the food being only available during darkness to being available 24 hr per day. After day 8 of manipulation the 24AL and 12AL groups did not differ for the remainder of the experiment (Figure 1) and averaged 36.4 ± 0.5^°^C in the 24AL group and 36.5 ± 0.3^°^C in the 12AL group (GLM, F_(1, 1016)_ = 0.74, *p* = 0.712). There was however a significant effect of day of measurement (GLM covariate, F_(1, 1016)_ = 20.27, *p* < 0.0005) and a significant interaction between the group and measurement day (F_(2, 1016)_ = 5.5, *p* = 0.019). There was a significant decline in temperature over the time course of the experiment in both groups, but this decline was steeper in the 24AL animals (gradient −0.044^°^C per 10 days compared with −0.013^°^C per 10 days in the 12AL animals) (Figure 1).

**Figure 2:**
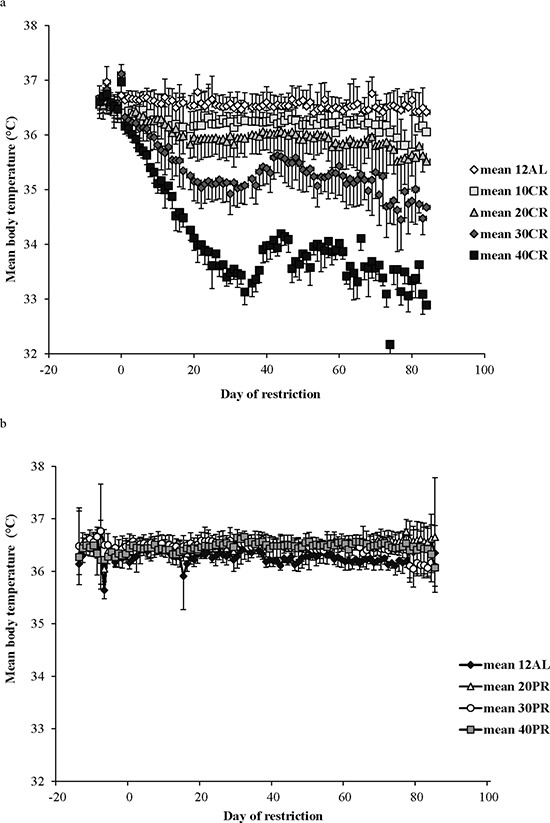
Mean daily body temperatures of the graded calorie restriction (CR) and protein restriction (PR) groups throughout the experiment **a.** The four CR groups, 10%, 20%, 30% and 40% restriction are labelled 10CR, 20CR, 30CR and 40CR respectively, along with the 12AL group fed *ad libitum* in 12 hrs of darkness. The 24AL group is omitted for clarity. **b.** The three PR groups, labelled 20PR, 30PR and 40PR, matched levels of protein for 20CR, 30CR and 40CR without a reduction in calories. The x-axis is the day of restriction with Day 0 the start of ‘calorie restriction’. Prior to that (negative days) all mice were at baseline and fed only during the 12 hrs of darkness. (*n* = 6 to 8 individuals per group). Note the different body temperature axis on this plot compared with Figure 1. Results are expressed as mean ± SD.

**Figure 3:**
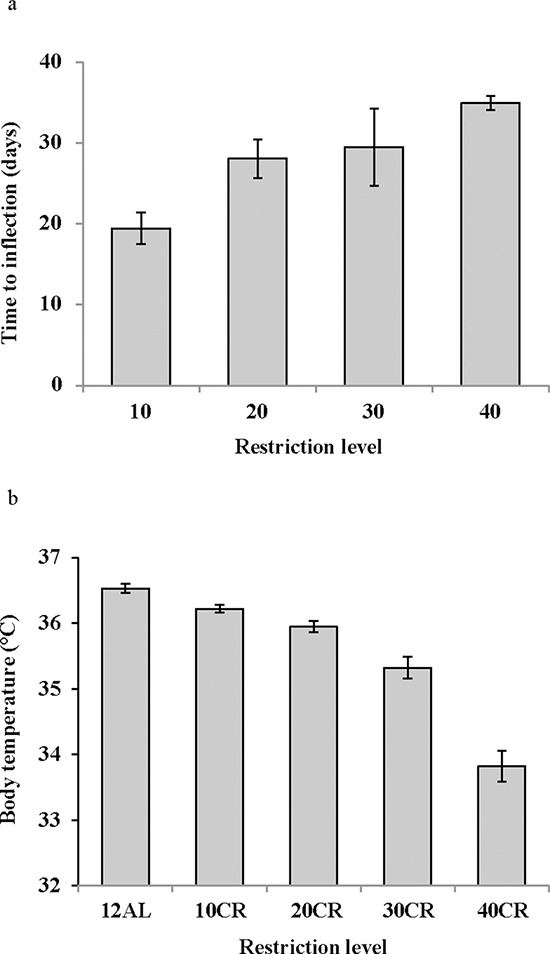
The response of mean daily body temperature to calorie restriction (CR) **a.** the time taken for the mean daily body temperature curve to inflect following the onset of restriction, calculated from the fitted polynomial regression curves (for curve parameters refer to Table 1), in relation to the level of restriction (10, 20, 30 and 40% CR) (*n* = 6 to 8 individuals per group). **b.** the average level of mean daily body temperature across all individuals between days 35 and 70 when the level was relatively stable.

In contrast to the shallow linear effect of time over the experimental period in the two *ad libitum* fed groups (Figure 1) there was a strong significant non-linear effect of time on the mean daily body temperature in all the groups exposed to CR (10%, 20%, 30% and 40% restricted food intake denoted as 10CR, 20CR, 30CR, and 40CR respectively) (Figure 2a). All of the animals on restriction showed an elevated body temperature on the first day that the food was restricted. This was followed by a progressive decline over the following period of up to 40 days followed by a slight increase. We fitted polynomial curves to the individual patterns of temperature change over the initial 40 days and in all but 3 cases there was a significant fit to a second order polynomial. The parameters of these fits are presented in Table 1. We calculated the inflection points of the fitted curves from the coefficient with respect to x divided by minus twice the coefficient in *x*^2^ (i.e. the solution of the differential of the polynomial for *x* = 0). For three animals on 10CR we could not fit curves because the body temperatures of these animals did not decline during this initial phase. Excluding these three animals the time taken to reach the inflection point was positively related to the level of restriction (Figure 3a) (F_(3, 23)_ = 9.21, *p* < 0.001). On average the animals on 10CR took 19.4 days to reach the nadir but the animals on 40CR took on average almost twice as long at 35 days. Following the nadir there was a slight increase in daily mean body temperature which lasted around 5 days in all groups. Body temperatures were then relatively stable until day 70. The average level of daily mean body temperature during this period of stability was significantly related (*p* < 0.001) to the extent of restriction (Figure 3b). Following day 70 there was a further period of decline in body temperature among the groups under restriction which lasted until the end of the experiment at day 84. This decline was not observed in the animals under *ad libitum*. The decline was linear and the gradient of the decline was positively related to the level of restriction. The body temperature of animals under greater restriction declined more during this final phase (gradients for each group for fitted linear regressions over last 15 days were 12AL = −0.0014, 10CR = −0.0158, 20CR = −0.0188, 30CR = −0.0281 and for 40CR = −0.0243).

**Table 1:** Parameters of fitted quadratic equations

Group	ID	a	b	c	int	*r*^2^
**10CR**	8	0.00297	−0.106	36.619	17.85017	0.772
	9	0.0044	0.15174	36.9075	17.24318	0.899
	21	0.00074	0.02538	36.408	−	0.08
	33	0.00041	0.00742	36.533	−	0.052
	46	0.00074	0.02892	36.489	19.54054	0.114
	50	0.00094	0.03855	36.61	20.50532	0.155
	54	0.00109	0.04805	36.72	22.04128	0.285
	56	0.00037	0.02724	36.716	−	0.028
	**Mean**				**19.44 ± 1.954**	
**20CR**	4	0.00042	0.02708	36.613	32.2381	0.573
	10	0.00127	0.06935	36.63	27.30315	0.812
	27	0.00089	−0.0538	36.502	30.22472	0.783
	37	0.00066	0.03574	36.65	27.07576	0.281
	39	0.0004	−0.0195	36.673	24.375	0.097
	47	0.00093	0.05015	36.512	26.96237	0.423
	57	0.0013	0.07133	36.8151	27.43462	0.63
	64	0.00115	0.06661	36.389	28.96087	0.628
	**Mean**				**28.07 ± 2.381**	
**30CR**	6	0.00281	0.14281	36.679	25.41103	0.814
	24	0.00178	0.08805	36.594	24.73315	0.843
	36	0.00082	0.06029	36.617	36.7622	0.732
	49	0.00251	0.13376	36.741	26.64542	0.744
	52	0.00318	0.19288	37.002	30.32704	0.741
	53	0.0011	0.07258	36.69	32.99091	0.722
	**Mean**				**29.48 ± 4.758**	
**40CR**	7	0.00367	0.19159	36.61	26.10218	0.867
	28	0.00348	0.23502	36.65	33.76724	0.893
	30	0.00257	0.17821	36.59	34.67121	0.811
	34	0.00242	0.19787	37.23	40.88223	0.779
	44	0.00117	−0.1208	36.71	51.62393	0.758
	48	0.0034	0.22284	36.88	32.77059	0.829
	58	0.00344	0.19393	36.31	28.1875	0.701
	62	0.00298	0.18842	36.87	31.61409	0.801
	**Mean**				**34.95 ± 8.06**	

y = a.x^2^ + b.x + c where y is the body mass, x is the day of restriction and a, b and c are arbitrary constants. Curves were fitted over the first 40 days of restriction, for individual mice (ID numbers) at 4 different restriction levels (10, 20, 30 and 40% signified as 10CR, 20CR, 30CR and 40CR respectively). The *r*^2^ of the fitted quadratic equation is shown along with the calculated interpolation point in days (int). Calculations were not made where the quadratic fit was not significant. Mean interpolation points ± SD are calculated for each group.

**Figure 4:**
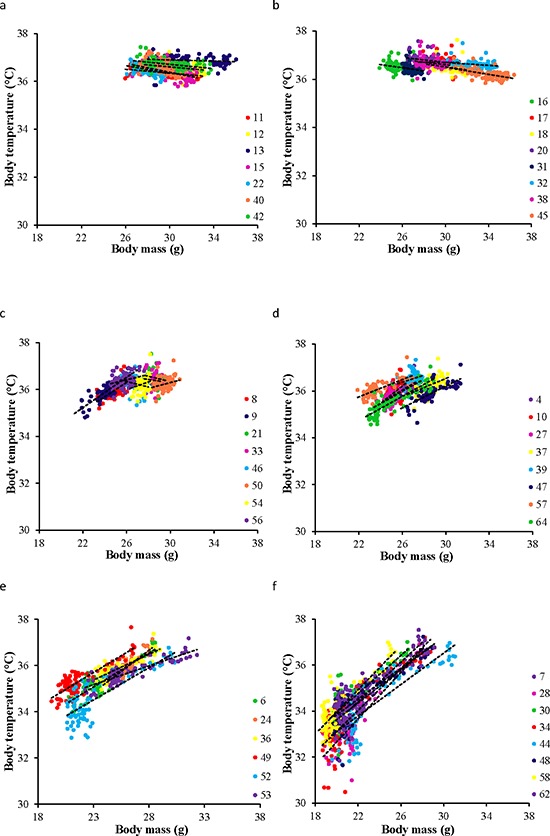
The relationship between body mass (BM) and mean daily body temperature throughout the entire experiment Individuals in each group are coded with a different symbol. 24AL and 12AL fed *ad libitum* for 24 and 12 hrs respectively **a** & **b.** The 4 treatment groups were restricted by 10, 20, 30, 40% referred to as 10CR, 20CR, 30CR and 40CR respectively **c–f.** In all groups there was a significant effect of BM on mean body temperature, a random effect of individual, and also an individual by mass interaction (for detailed statistics refer to Table 2). In the AL fed groups BM was negatively related to body temperature and in the CR fed animals it was positively related to body temperature.

**Figure 5:**
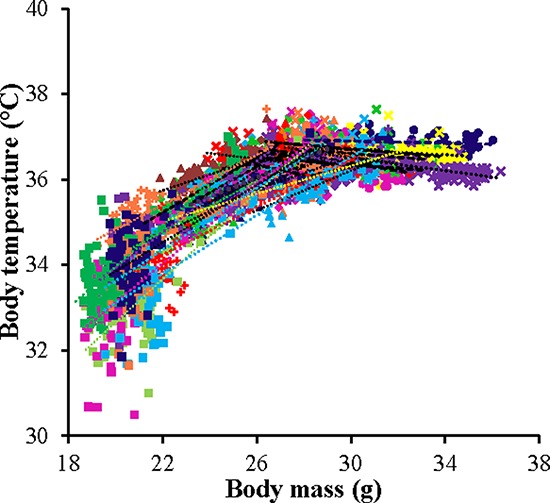
The relationship between mean daily body temperature and body mass (BM) Data was pooled data across all individuals in all treatment groups (*n* = 4126). Individuals are coded with different symbols. There appeared to be a pivotal BM of 27g above which mass had a negative effect on body temperature and below which the effect was positive.

There were some similarities and differences in these patterns to the simultaneously recorded changes in BM [43]. BM also declined during the initial phase of restriction and reached a nadir about 30 days later. However, in contrast to the change in body temperature the time to reach the minimum BM was independent of the level of restriction. BM (and body fatness/lean mass) also remained constant after this initial 30 day dynamic phase until the end of the experiment and there was no indication of a secondary phase of modulation during the last 15 days of the manipulation—as occurred with body temperature. Despite this, there was a significant relationship between BM and mean daily body temperature within each of the restriction groups throughout the duration of the experiment (Figure 4). In the two *ad libitum* groups there was a significant negative relationship between BM and body temperature (Figure 4a and 4b) with a significant random effect of individual, and a significant individual by BM interaction (Table 2). Hence, the individuals did not sit on a common line, or have a common negative gradient. Within each of the restriction groups the pattern was similar in that there were significant effects of BM and individual and a significant BM by individual interaction (Table 2) but in these groups the relationship of BM to body temperature was positive (Table 2 and Figure 4c–4f). Pooling all the data across all the individuals revealed that there was a peaked relationship between daily records of BM and body temperature (Figure 5). Above a BM of 27 g the relationship between temperature and BM was slightly negative. Within the data cloud in this region the individual mice had negative relationships between BM and body temperature. Below 27 g there was a positive curvilinear relationship between temperature and BM. In this zone the individuals had positive relationships between BM and body temperature (Figure 5). Hence while the detailed pattern of the time courses of the relationships between BM and body temperature differed, they did so within a broad general relationship between the two variables.

**Table 2:** Significant parameters influencing mean daily body temperature

Source	df	F	*p*
**24AL**			
Individual	6	3.8	0.001
Body mass	1	101.27	<0.0005
BM*ID	6	5.31	<0.0005
Error	574		
**12AL**			
Individual	7	75.44	<0.0005
Body mass	1	46.71	<0.0005
BM*ID	7	2.01	NS
Error	661		
**10CR**			
Individual	7	19.31	<0.0005
Body mass	1	32.47	<0.0005
BM*ID	7	20.06	<0.0005
Error	670		
**20CR**			
Individual	7	4.32	<0.0005
Body mass	1	322.7	<0.0005
BM*ID	7	2.92	0.005
Error	672		
**30CR**			
Individual	5	13.55	<0.0005
Body mass	1	905.2	<0.0005
BM*ID	5	13.41	<0.0005
Error	508		
**40CR**			
Individual	7	6.49	<0.0005
Body mass	1	1560.5	<0.0005
BM*ID	7	5.89	<0.0005
Error	672		

GLM analyses including factors that influence mean daily body temperature at each level of restriction. The factors included in the models were daily body mass (BM), individual and the individual by BM interaction (BM*ID).

**Figure 6:**
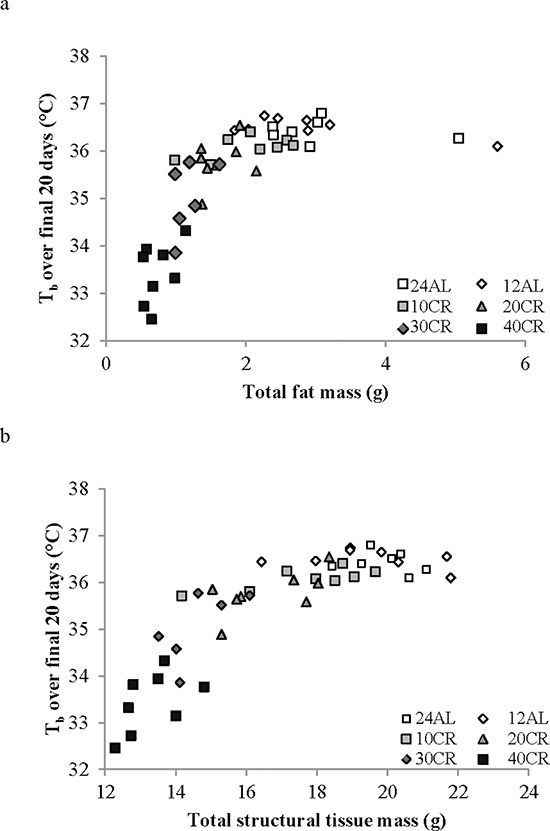
The relationship between mean daily body temperature (T_b_) measured over the last 20 days of the experiment and body composition T_b_ was averaged over days 65–84 and plotted against the body composition of the animals at dissection (day 84). Data on body composition are from [43]. **a.** the effect of total body fat (pooled masses of sub-cutaneous, epididymal, retroperitoneal and mesenteric fat depots). **b.** the effect of total structural tissue (pooled carcass, skin and tail masses). (Please see text for statistical details).

**Figure 7:**
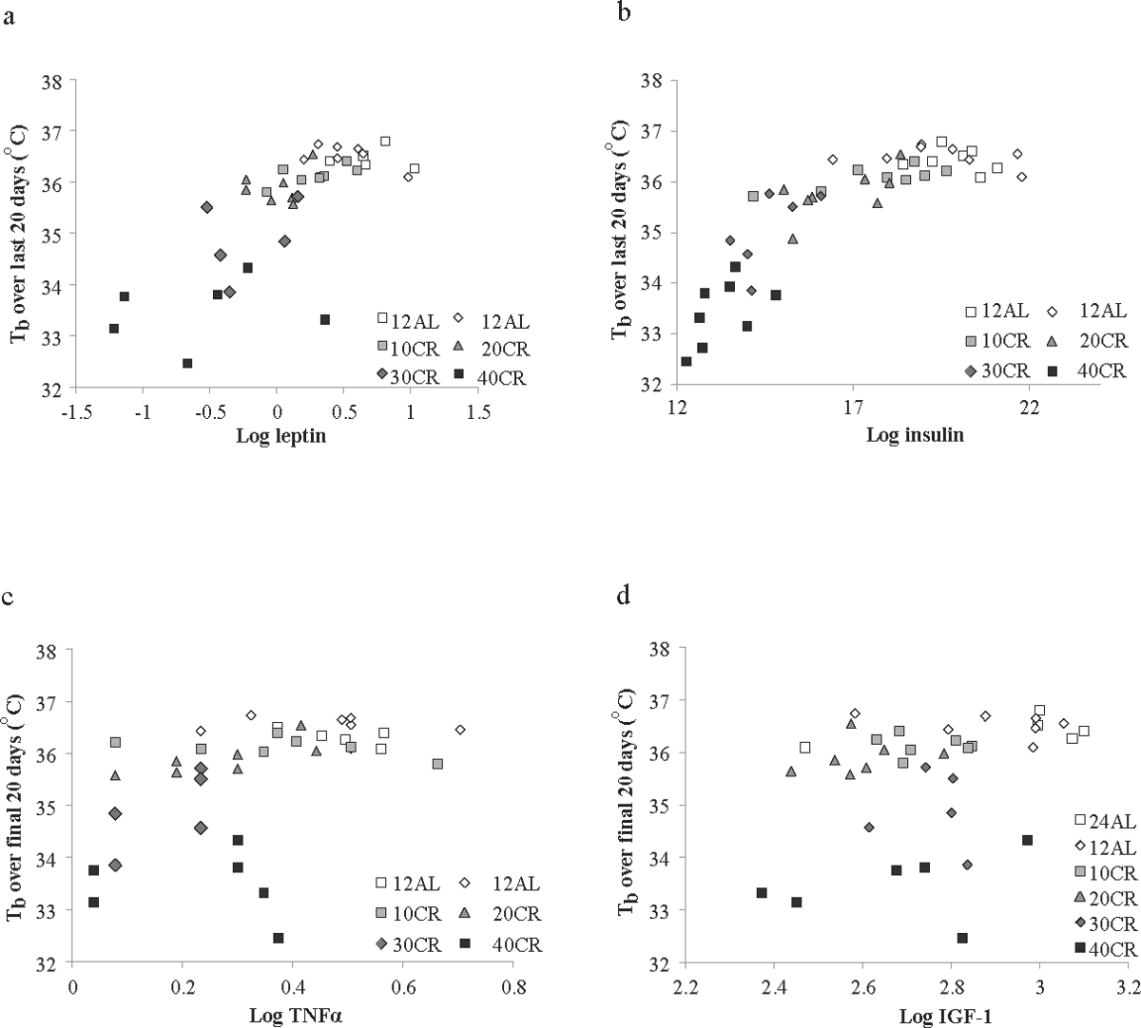
The relationship between mean daily body temperature (T_b_) measured over the last 20 days of the experiment and hormone levels T_b_ was averaged over the last 20 days 65–84 and plotted against circulating hormone levels measured at dissection (day 84). Data on hormone levels were previously reported [44]. **a.** leptin **b.** insulin, **c.** tumor necrosis factor alpha (TNF-α) and **d.** insulin like growth factor 1 (IGF-1). In all cases there were significant effects (see text for statistical details).

Moreover, there was an extremely strong and significant relationship between the average body temperature over the final 20 days of manipulation and body composition of the mice based on dissection on the final day (data from [43]). Average body temperature was significantly non-linearly related to the total mass of dissected body fat (epididymal, retroperitoneal, subcutaneous and mesenteric fat depot sizes pooled) (Figure 6a: F_(2, 42)_ = 66.15, *p* < 0.0001, *r*^2^ = 0.759 for quadratic fit) and significantly nonlinearly related with the total mass of structural tissue (pooled mass of carcass, skin and tail) (Figure 6b: F_(2, 42)_ = 113.69, *p* < 0.0001, *r*^2^ = 0.844 for quadratic fit). There was also a significant interaction between these effects (F_(1, 41)_ = 48.0, *p* < 0.0001 for interaction effect in GLM). Together fat tissue, structural tissue and their interaction explained 85.1% of the variance in the body temperature over the last 20 days of the manipulation. There was no significant relationship between body temperature and the pooled mass of the vital organs (F_(1, 41)_ = 1.21, *p* > 0.05), alimentary tract components (F_(1, 41)_ = 0.25, *p* > 0.05) or the mass of the reproductive tissue (F = 0.76, *p* > 0.05). We also examined the relationships between circulating hormone levels at the end of the experiment [44] and the average body temperature over the last 20 days of the experiment. There was a significant relationship between body temperature and circulating leptin levels (Figure 7a: log leptin F_(1, 37)_ = 44.99, *p* < 0.0005), circulating insulin levels (Figure 7b: log insulin F_(1, 36)_ = 4.73, *p* = 0.036), circulating tumor necrosis factor alpha levels (Figure 7c: log TNF-α F_(1, 37)_ = 10.39, *p* = 0.003) and circulating IGF-1 levels (Figure 7d: log IGF-1 F_(1, 39)_ = 23.42, *p* < 0.0005). The association to levels of resistin (log resistin F_(1, 36)_ = 1.58, *p* < 0.217) and interleukin-6 (log IL-6: F_(1, 36)_ = 0.14, *p* = 0.714) were both not significant. In a multiple regression only the effects of leptin and IGF-1 remained significant and together these two variables explained 60.2% of the variation in body temperature during the last 20 days of the experiment (combined F_(2, 37)_ = 26.46, *p* < 0.0001).

Inspection of the time course of the variation in body temperature (Figure 2a) suggested that once the initial decline in temperature was over there might be cycles of change over the remaining period. We therefore explored this possibility by calculating autocorrelation and partial autocorrelation functions for the mean daily body temperatures between days 35 and 85 for each group (supplementary Figure 1). These autocorrelation and partial autocorrelation functions suggested that there were strong correlations between days only with a lag of a single day. There was no evidence of any longer term cycles in the daily body temperatures.

**Figure 8:**
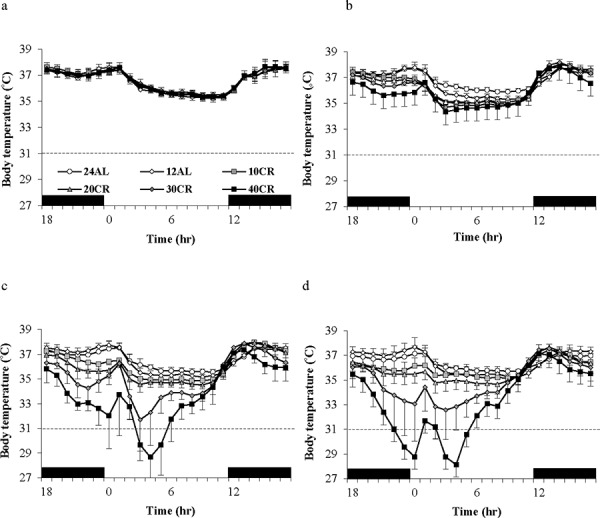
Body temperature fluctuations over a 24 hr period at varying timepoints over 12 weeks of calorie restriction (CR) **a.** baseline, **b.** 1 week of CR **c.** 4 weeks of CR and **d.** at the end of study following 11 weeks of CR. The period of darkness is shown by black bars along the x-axis. CR mice were fed at lights out. 24AL and 12AL represent animals fed *ad libitum* for 24 and 12 hrs respectively. The 4 treatment groups restricted by 10, 20, 30, 40% are referred to as 10CR, 20CR, 30CR and 40CR respectively. Data is presented as mean ± SD.

Daily body temperature fluctuations over 4 time points of the 12 weeks of the study are shown in Figure 8a–8d. Over baseline body temperatures were similar across the groups (GLM-RM: diet F_(5, 40)_ = 1.83, *p* = 0.128). However the fluctuations were significant over the 24 hours (GLM-RM: time F_(23, 920)_ = 449.11, *p* < 0.0005) with body temperature naturally lowered in the non-active light phase (Figure 8a). The peak in body temperature around 0630 is coincident with the removal of food from the 12AL. As early as week 1 changes in body temperatures were evident (GLM-RM: diet F_(5, 40)_ = 4.78, *p* = 0.002; time F_(23, 920)_ = 253.80, *p* < 0.0005, diet X time interaction (F_(115, 920)_ = 1.76, *p* < 0.0005)) (Figure 8b). Compared to the 12AL animals the body temperature of 30 and 40CR groups were significantly lower (post hoc Tukey *p* = 0.007 and *p* = 0.001 respectively). By week 4, reflective of the time taken to reach inflection point of the 30 and 40CR groups (Figure 3a), clear differences in the pattern of daily body temperatures were observed (GLM-RM: diet F_(5, 40)_ = 62.46, *p* < 0.0005), time (F_(23, 920)_ = 129.60, *p* < 0.0005) with a strong interaction between the 2 parameters (F_(115, 920)_ = 11.06, *p* < 0.0005) (Figure 8c). Post-hoc Tukey tests found, in addition to the 30CR (*p* < 0.0005) and 40CR (*p* < 0.0005) groups, body temperatures of the 20CR (*p* < 0.05) were also significantly lower than that of the 12AL with minimum temperatures of 35.5 ± 1.85^°^C, 31.8 ± 2.36^°^C and 28.64 ± 1.97^°^C at 1400, 0930 and 1030 respectively. The diet (GLM-RM: diet F_(5, 40)_ = 49.51, *p* < 0.0005), time (F_(23, 920)_ = 56.90, *p* < 0.0005) and interaction (F_(115, 920)_ = 11.54 *p* < 0.0005) remained significant at 11 weeks (Figure 8d). Mice in the 40CR group further lowered their body temperature (28.15 ± 0.45^°^C) while there was a slight increase in body temperature of the 30CR group (32.55 ± 0.55^°^C). The body temperature of these two groups was significantly lower than all other groups (post hoc Tukey *p* < 0.008). The mean daily temperatures of these 4 time points are shown in Table 3a.

**Table 3:** Mean daily body temperatures at 4 timepoints recorded over 12 weeks

a) graded levels of calorie restriction (10, 20, 30 and 40% CR). Data presented as mean ± SD				
Group	BL	1 week	4 week	11 week
24AL	36.6 ± 0.15	36.9 ± 0.14	36.7 ± 0.19	36.5 ± 0.24
12AL	36.6 ± 0.14	36.7 ± 0.11	36.6 ± 0.24	36.5 ± 0.22
10CR	36.7 ± 0.08	36.4 ± 0.11	36.3 ± 0.30	36.0 ± 0.31
20CR	36.6 ± 0.21	36.3 ± 0.15	36.1 ± 0.40	35.8 ± 0.49
30CR	36.7 ± 0.17	36.1 ± 0.09	35.1 ± 0.76	34.9 ± 0.85
40CR	36.7 ± 0.17	35.9 ± 0.18	33.9 ± 0.50	33.2 ± 0.68

b) graded levels of protein restriction (20, 30 and 40% PR).				
Group	BL	1 week	4 week	11 week
12AL	36.3 ± 0.21	36.4 ± 0.12	36.3 ± 0.04	36.2 ± 0.05
20PR	36.4 ± 0.16	36.5 ± 0.03	36.5 ± 0.04	36.6 ± 0.06
30PR	36.5 ± 0.12	36.5 ± 0.02	35.6 ± 0.02	36.4 ± 0.01
40PR	36.3 ± 0.09	36.5 ± 0.03	36.5 ± 0.03	36.5 ± 0.04

#### Torpor use

We defined torpor as having occurred if, any hourly average was 31^°^C or lower (dashed line in Figure 8) [48]. Hence, by this criterion, the 40CR animal displayed torpor during this daily cycle, but the 24AL animal did not. We defined the torpor temperature as the average minute by minute temperature during the period spent below 31^°^C. In this case the torpor temperature for the 40CR animal was 27.5^°^C.

**Figure 9:**
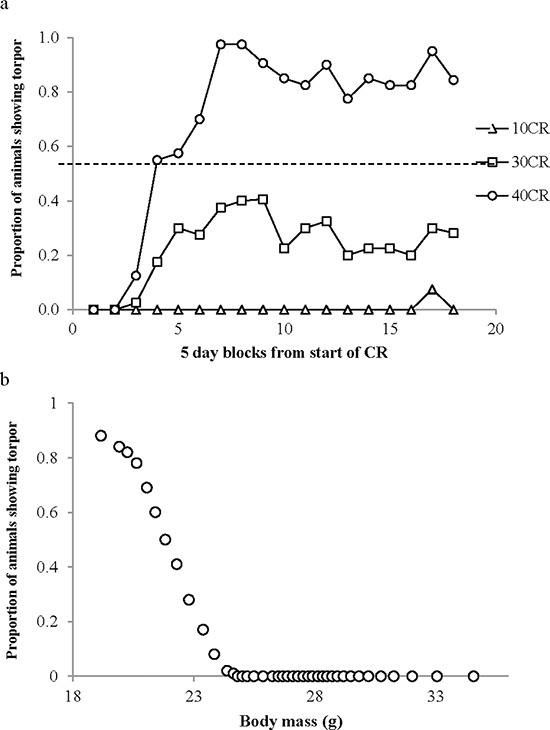
Torpor use during calorie restriction (CR) **a.** the proportion of animal-days when torpor was reported within the 3 treatment groups displaying torpor (10CR, 30CR and 40CR. The x-axis shows the time course of the experiment split into 5 day periods. **b.** The probability of an individual animal showing torpor in relation to body mass, calculated across all 4126 animal-days.

We split the time course of the experiment into 5 day periods and then counted the number of days that the animals in each treatment group went torpid and expressed this as a proportion of the total potential torpid animal-days (Figure 9a). Torpor was only observed in the 40CR and 30CR groups, and on 3 occasions in the 10CR group, but was never observed in the 20CR, 12AL and 24AL groups. No animals were observed torpid during the baseline period or during the first 5 days on restriction. The first torpid animal was observed on day 8 in the 40CR group. The proportion of days when torpor was observed increased steadily in the 40CR animals and to a lesser extent in the 30CR animals until during days 26 to 35 it was observed on 97.5% of possible occasions for the 40CR group and between days 31 to 40 it was observed on 40% of possible occasions for the 30CR animals. Thereafter the frequency of occurrence declined slightly in both groups averaging 80–90% of occasions in the 40CR animals and 20–30% of occasions in the 30CR animals. A single animal in the 10CR group had 3 days of torpor between days 75–80. In total across 4126 animal days of observation there were 604 days with torpor (14.64%) or 15.85% if the baseline days are excluded. BM was a strong predictor of the probability of entering torpor (binary logistic regression: BMz = 23.3, *p* < 0.0001). We divided the data cross all individuals ordered by BM into batches of 100 samples (*n* = 41) and in each batch calculated the proportion of days including torpor and the average BM (Figure 9b). No mice with BM greater than 24.8 g ever entered torpor. The proportion entering torpor increased dramatically at lower BMs. The pivotal BM, where 50% of occasions involved torpor, was 21.8 g. Below a BM of 20 g torpor occurred on >80% of days but at no mass was torpor observed on 100% of occasions.

**Figure 10:**
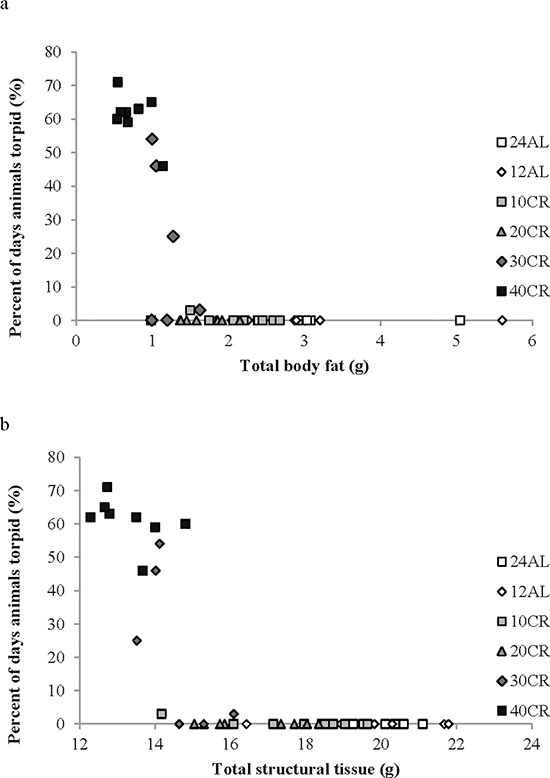
The effect of body composition on the propensity of individual animals to display torpor **a.** body fat and **b.** structural tissue. The maximum possible number of torpor days during the experiment was 84. Different groups are shown with different symbols.

We explored the individual characteristics at the end of the experiment that were associated with the display of torpor in the individual animals. The maximum number of days that any animal displayed torpor was 71 out of 84 possible treatment days for one of the 40CR animals. Torpor occurrence (n days) was negatively related to the level of body fat (F_(2 42)_ = 37.04, *p* < 0.0005, *r*^2^ = 0.638 for quadratic fit: Figure 10a), the level of structural tissue (F_(2, 42)_ = 82.80, *p* < 0.0005, *r*^2^ = 0.798 for quadratic fit: Figure 10b) and the total mass of the reproductive organs (F_(2, 42)_ = 17.66, *p* < 0.0005, *r*^2^ = 0.457 for quadratic fit). The single animal in the 10CR group that showed 3 days of torpor near the end of the experiment had the lowest level of structural tissue, and the second lowest level of fat tissue, of all the animals in the 20CR, 10CR, 12AL and 24AL groups. Among the circulating hormone levels the occurrence of torpor was significantly related to, the circulating levels of leptin (lower levels more torpor, log leptin F_(1, 36)_ = 28.02, *p* < 0.0005), TNF-α (lower levels more torpor, log TNF-α F_(1, 36)_ = 7.29, *p* = 0.011) and IGF-1 (lower levels more torpor, F_(1, 39)_ = 14.92, *p* < 0.0005) but the relationships with insulin (F_(1, 36)_ = 2.23, *p* = 0.144), IL-6 (F_(1, 36)_ = 0.39, *p* = 0.536) and resistin (F_(1, 36)_ = 1.56, *p* = 0.222) were not significant. When including all the hormone levels together in a multiple regression analysis only leptin and IGF-1 were significant (combined F_(2, 35)_ = 17.50, *p* < 0.00005, *r*^2^ = 0.500).

**Figure 11:**
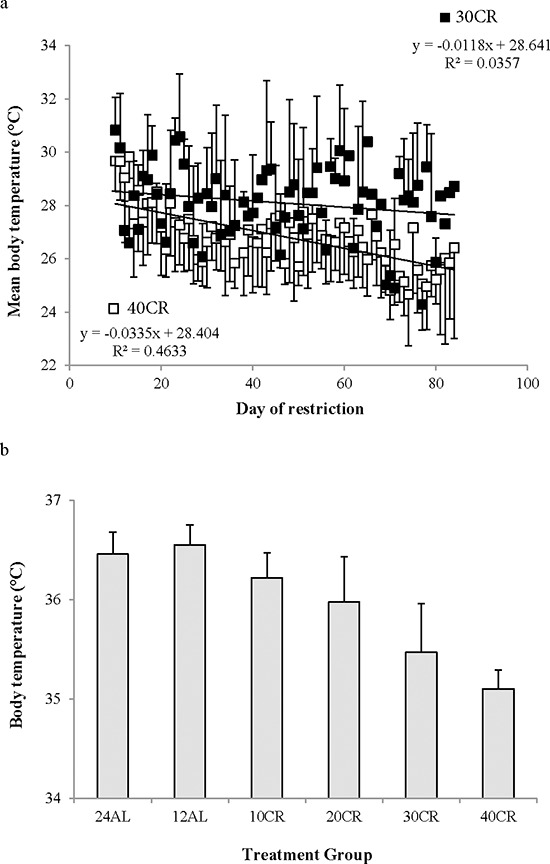
Body temperature during torpid and non-torpid bouts **a.** the average body temperature during torpor bouts in relation to the day of restriction for mice under 30% calorie restriction (30CR) (closed symbols) and 40CR (open symbols). Mice went into deeper torpor when exposed to 40CR compared to 30CR and torpor temperature declined as the experiment proceeded. **b.** non-torpid body temperature over the last 20 days of the manipulation (days 65 to 84) calculated as the temperature averaged across hourly periods when the body temperature was greater than 31^°^C. Data are presented as mean ± SD.

The average temperature during torpor declined significantly over the measurement period (F_(1, 144)_ = 20.29, *p* < 0.001). The first displays of torpor included average temperatures around 29–30^°^C, but the final torpor bouts included average temperatures that were about 3^°^C cooler than this at 26–27^°^C. The rate of decline was significantly different between the 30CR and 40CR animals (Figure 10: group by day interaction F_(1, 144)_ = 6.76, *p* = 0.015) but the main group effect was not significant. The mean temperature during torpor in the 40CR group was 26.8 ± 1.1^°^C compared with 28.1 ± 1.4^°^C in the 30CR group (Figure 11a). The mean body temperature when not torpid averaged across the last 20 days of the manipulation was strongly related to the level of restriction (One way ANOVA F_(5, 40)_ = 25.35, *p* < 0.0005) with those under greater levels of restriction having lower non-torpid body temperatures (Figure 11b). On average the AL animals had non-torpid body temperatures of 36.5 (0.22)^°^C for 24AL and 36.6 (0.20)^°^C for 12AL (nsd *p* > 0.05 by Tukey test), but the means for the restricted animals were 36.2 (0.25)^°^C for 10CR, 36.0 (0.45)^°^C for 20CR, 35.5 (0.49)^°^C for 30CR and 35.1 (0.19)^°^C for those under 40CR. The overall lower body temperature in mice under the highest levels of restriction was therefore due to a combination of increased time spent in torpor and lower body temperature both when in and not in torpor.

### Protein restriction (PR)

#### Mean daily body temperature

**Figure 12:**
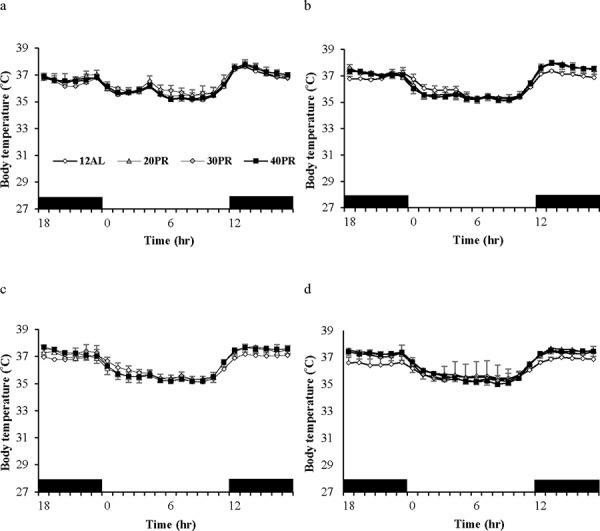
Body temperature fluctuations over a 24 hr period at varying timepoints over 12 weeks of graded protein restriction (PR) throughout the experiment Body temperatures were measured in the three restricted protein intake groups (20%, 30% and 40% restriction labelled 20PR, 30PR and 40PR respectively) along with the control group fed 12 hrs *ad libitum* (12AL) at **a.** baseline, **b.** 1 week, **c.** 4 week and **d.** 11 weeks of study. The x-axis is the day of measurement. Day 0 is the start of PR. Prior to that (negative days) all mice were at baseline and fed only during the 12 hours of darkness. (*n* = 8 individuals per group). Data is presented as mean ± SD.

The mean daily body temperature over the baseline period across all the mice in the PR groups was 36.3^°^C (SD = 0.14, *n* = 17 individuals measured over 14 days; Figure 12a). There was no significant group difference over this period (F_(3, 183)_ = 0.89, *p* = 0.47). Over the entire experiment there was a non-significant effect of group (F_(3, 1704)_ = 2.81, *p* = 0.075) but a highly significant group by day interaction (F_(3, 1704)_ = 22.89, *p* < 0.0005). Day of measurement was not significant (F_(1, 1704)_ = 0.02, *p* = 0.897). Using the daily means across individuals there was a significant effect of group (GLM-RM, F_(3, 403)_ = 19.52, *p* < 0.0005 and a significant group by day interaction F_(3, 403)_ = 29.01, *p* < 0.0005, but no overall significant effect of day (F_(1, 403)_ = 0.63, *p* = 0.428). Tukey pairwise post hoc comparisons revealed that the 40PR group had significantly higher mean daily body temperature than the other 3 groups, but no other pairwise differences were significant. Over time the mean daily body temperature of the 40PR mice significantly increased (Figure 2b) (mean daily body temperature = 36.412 + 0.0023 Day, F_(1, 101)_ = 38.71, *p* < 0.0005, *r*^2^ = 0.279). In contrast there was a small but significant decline in the 30PR animals (regression: mean daily body temperature = 36.527 −0.000785 Day, F_(1, 101)_ = 6.39, *p* = 0.013, *r*^2^ = 0.060), and no significant relationship in the 20PR group (regression: mean daily body temperature = 36.441 −0.000151 Day, F_(1, 101)_ = 0.14, *p* = 0.713, *r*^2^ = 0.001). In the 12AL animals there was a more significant decline during the period of the experiment (regression: mean daily body temperature = 36.344 −0.00223 Day, F_(1, 101)_ = 51.61, *p* < 0.0005, *r*^2^ = 0.336) (Figure 2b). The mean daily temperatures of mice under PR over the course of the study are shown in Table 3b.

#### Torpor use

In contrast to mice on CR (Figure 8a–8d) the daily fluctuations in body temperatures of the PR mice remained similar to that of baseline over the 12 weeks of PR (Figure 12a–12d). No animals in the PR or PR-12ALgroups were observed to enter torpor during the entire experiment.

### Comparison of responses to calorie and protein restriction (CR vs PR)

**Figure 13:**
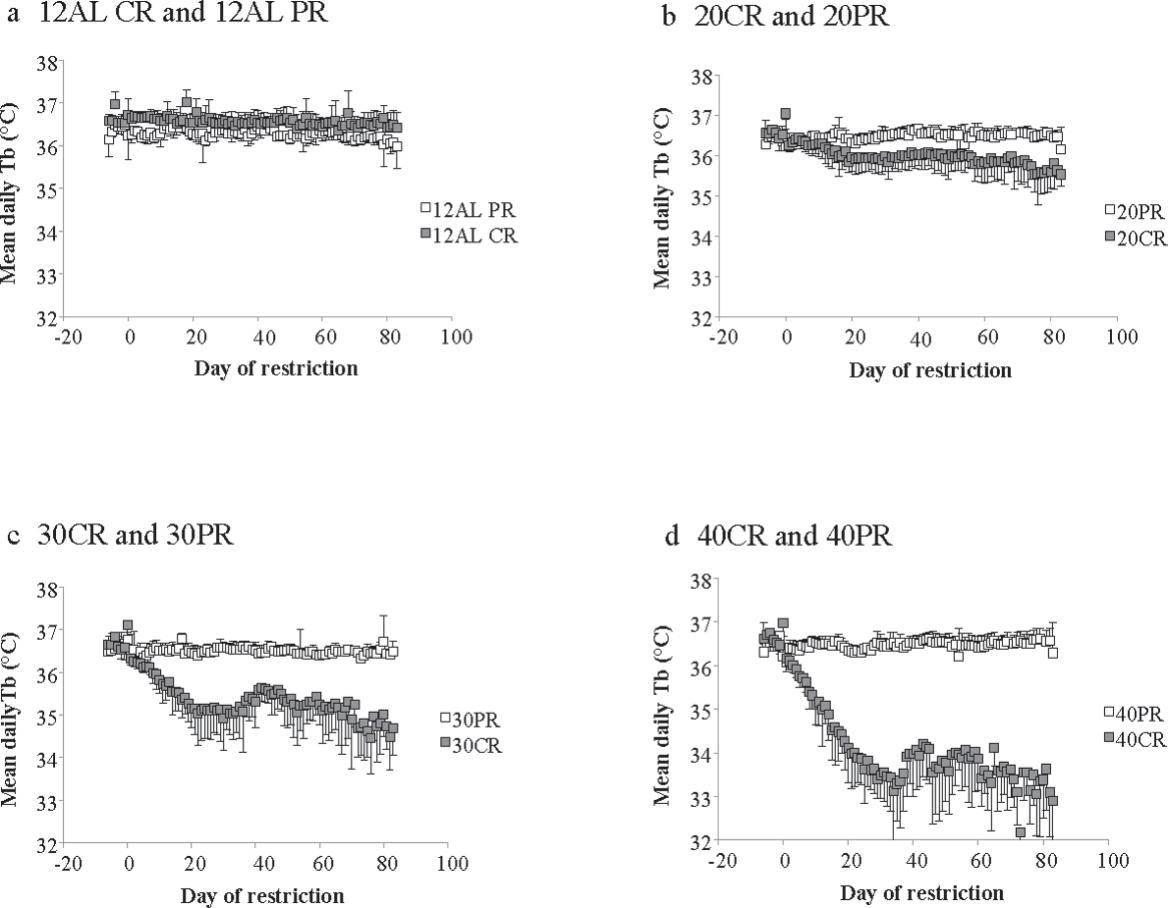
Comparisons of the body temperature responses to caloric restriction (CR) and protein restriction (PR) **a.** the two control *ad libitum* fed groups (12AL) that were both fed the same diet (20% protein) available only during the hours of darkness. **b**, **c.** and **d.** show the mice under respectively 20, 30 and 40% CR matched to mice under 20, 30 and 40% PR. All plots are presented on a common scale. The x-axis is the day of measurement. Day 0 is the start of restriction. Prior to that (negative days) all mice were at baseline and fed only during the 12 hours of darkness. (*n* = 8 individuals per group except in 30CR where *n* = 6). Data is presented as mean ± SD.

Since protein content of the PR diets were designed to match the protein intake of the nominal 20, 30 and 40% CR animals, we compared the body temperature responses of the CR groups to the equivalent PR animals to establish the extent to which the impact of CR might be attributable to the reduced levels of protein in the CR diet. In both experiments there was a 12AL group fed 20% protein diet which were treated identically throughout the treatments. Despite this identical treatment these groups differed in their body temperatures throughout the overall experiment (F_(1, 1067)_ = 12.55, *p* = 0.005) with the PR-12AL animals having significantly lower body temperatures than the CR-12AL group (Figure 13a) by about 0.3^°^C. Despite this difference there were also broad similarities in the way the two groups responded over the duration of the treatment. Hence, both 12AL groups declined in body temperature throughout the experiment (Day effect: F_(1, 1067)_ = 20.04, *p* < 0.005) and the gradient of decline was almost identical between the two groups (−0.0022 in the CR-12AL animals and −0.0021 in the PR-12AL animals) with the group by day interaction not significant (F_(1, 1067)_ = 2.03, *p* = 0.37: Figure 13a).

Although the CR-12AL and PR-12AL groups differed significantly at the start of the experiment during baseline (F_(1, 100)_ = 24.14, *p* < 0.0001) this was not true for the 20CR vs 20PR and 30CR vs 30PR comparisons (20CR v 20PR: GLM-RM: group effect at baseline F_(1, 100)_ = 3.9, *p* = 0.074; 30CR vs 30PR : group effect at baseline F_(1, 87)_ = 4.47, *p* = 0.061) However for the 40CR and 40PR groups there was also a baseline difference with the 40CR group having a significantly higher body temperature than the 40PR animals; 40CR vs 40PR group effect at baseline F_(1, 72)_ = 10.47, *p* < 0.001). The responses of the 20CR and 20PR to the treatment were significantly different (Figure 13b). There was a large day effect on mean body temperature (GLM-RM F_(1, 1187)_ = 244.01, *p* < 0.0005) and a large random effect of individual (F_(11, 1187)_ = 98.1, *p* < 0.0005) and a large day by group interaction (F_(1, 1187)_ = 264.31, *p* < 0.0001) reflecting the almost constant level of mean temperature in the 20PR animals, and the significant decline in those under 20CR (Figure 13b). These effects and the significances of the differences were magnified in the 30CR vs 30PR (Figure 13c) and 40CR vs 40PR comparisons (Figure 13d). Hence for the 30CR vs 30PR groups there was a large day effect on mean body temperature (GLM-RM F_(1, 907)_ = 397.3, *p* < 0.0005), a large random effect of individual (F_(8, 907)_ = 49.97, *p* < 0.0005) and a large day by group interaction (F_(1, 907)_ = 205.59, *p* < 0.0001). Finally for the 40CR animals compared with the 40PR group (Figure 12d) there was a large day effect on mean body temperature (GLM-RM F_(1, 1085)_ = 394.4, *p* < 0.0005) a large random effect of individual (F_(10, 1085)_ = 20.53, *p* < 0.0005) and a large day by group interaction (F_(1, 1085)_ = 515.19, *p* < 0.0001), reflecting the fact the 40PR group increased temperature slightly over the treatment period while the body temperatures of the 40CR animals were dramatically reduced.

## DISCUSSION

Following the initiation of restriction all mice in the CR groups showed a progressive decline in mean daily body temperature below the baseline levels, until a new steady state was reached 17 to 35 days later (Table 1 and Figure 3). Although BM also declined over the same period the two were not closely coordinated. For example, the time for the body temperature to stabilize was strongly dependent on the level of restriction (Figure 3), but the time for the BM to stabilize was not [43]. Also in the latter stages of the experiment (days 70–84) the body temperature declined further (Figure 2a), but BM did not. Many previous studies have shown an impact of CR on body temperature [22, 24, 26, 45, 49–51] also see review by [52]. What is new in our study is the demonstration that the effect on body temperature, across graded levels of restriction, is progressive. Since lifespan extension is also linearly linked to the extent of CR, the parallel impact on body temperature, especially given the role it plays in a variety of physiological mechanisms, is consistent with suggestions that the reduced temperature may be an integral causal aspect of the CR effect [36, 52].

Despite asynchronicity in the way BM and body temperature changed with CR there was a broad consistency in both variables in tandem with CR levels (Figure 5). This broad linkage was exemplified by the association between terminal measures of body composition, hormone levels and mean body temperature over the last 20 days of restriction (Figures 6 and 7). Expression of torpor was also closely associated with the BM and body composition of the animals (Figures 8 to 11) being expressed only when BM fell below a critical level (Figure 9). Rikke and colleagues also observed that across mouse strains the body temperature under CR was lower in strains with lower BM [26]. In the mouse lemur, a small primate that uses torpor [53], expression of torpor in lactation was similarly only observed at the highest levels of restriction [54]. The effect of body composition on mean daily temperature, however, was more than an effect on torpor expression, because there was a relationship between BM and mean daily body temperature across the 12AL, 10CR and 20CR animals (Figure 3) in which torpor was not observed (apart from 3 reports in a single individual from the 10CR group which had exceptionally low adipose and structural tissue levels compared to others in these groups). Moreover, rats and non-human primates under CR that show a lifespan extension effect, do so without displaying torpor [6, 24].

Despite this correlation between the level of CR and mean body temperature, the pattern of change in body temperature over time suggests the body temperature responses were not directly driven by the shortfall in calorie intake relative to demand. That is, the shortfall in calorie intake would have been greatest on the first day of restriction and then would progressively get smaller as the animals energy expenditure declined, eventually falling to zero at the point where mass loss fell to zero (about 30 days). In contrast the body temperature declined slowly from day 1 of restriction and then stabilized at a low point after the shortfall had been eliminated.

**Figure 14:**
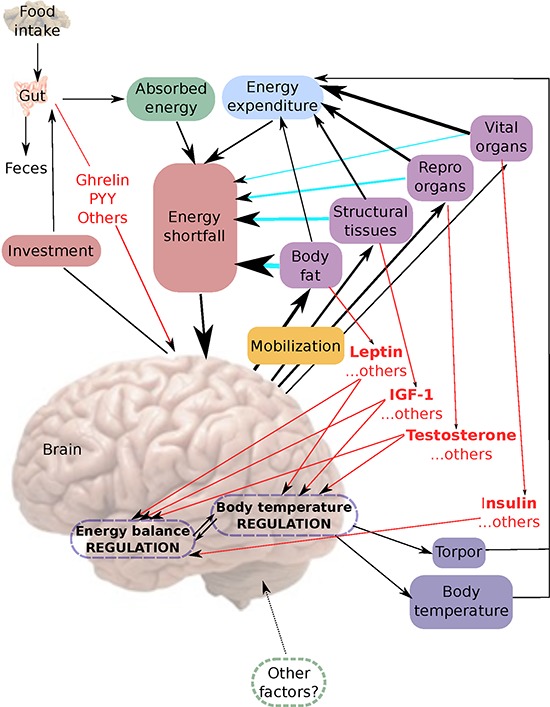
A hypothetical model for the interplay of body composition and body temperature in response to caloric restriction (CR) For further explanation refer to the text.

The data in the current and previous studies of these mice [43, 44] suggest a model for the body temperature response to CR as follows (Figure 14). Food is ingested into the alimentary tract and some of it is absorbed with the remainder eliminated as feces. The absorbed energy and the level of energy expenditure together define the level of energy shortfall. This shortfall is detected by the energy balance regulation system in the arcuate, dorsomedial and ventromedial nuclei of the hypothalamus. Exactly how this is signaled is unclear but gut hormones (in red Figure 14) may provide a mechanism for monitoring intake, and signals from the vital organs, reproductive organs, structural tissues and adipose tissues (also in red) may be instrumental in signaling energy expenditure. The response to the perceived energy shortfall is to invest in growth of the alimentary tract to elevate absorption efficiency [43, 55], at the same time as mobilizing the remaining body tissues. This mobilization happens in a hierarchical fashion [43] with the vital organs least and adipose tissue most favored for mobilization (arrows in Figure 14 indicate magnitude of mobilization). The mechanisms underlying this differential tissue utilization and investment are currently unknown. However, altered levels of SIRT in adipose tissue probably underlie a shift towards elevated lipolysis [56, 57] and hence reduced adipose tissue mass. The consequences of the tissue utilization are that energy derived from burning the tissues contributes to reducing the shortfall between intake and expenditure (blue arrows). Moreover the altered masses of the body components affect energy expenditure. The largest contributors to this effect are the vital and reproductive organs and structural tissue [58, 59] with fat playing a lesser role [59, 60]. The impact of fat may be mediated via the reduced levels of adipokines that then impact on lean tissue metabolism (e.g. [61]). This reduced energy expenditure also contributes to minimizing the shortfall.

The changes in the sizes of the adipose tissue and lean tissue compartments results in changes in the levels of circulating hormones produced by these tissues. We have shown that this includes reduced levels of leptin produced by adipose tissue, IGF-1 produced by skeletal muscle, and insulin produced by the pancreas [44]. Reductions in levels of reproductive hormones such as testosterone, which we did not measure, have been previously reported in rodents on 40–60% CR [62] including wild rodents that show no longevity benefits from CR [63]. Testosterone was not reduced with 20% CR [64], pointing to a graded effect of restriction. The effect of 20% CR on testosterone levels in non-human primates also appears minimal [65]. As it becomes increasingly clear that most tissues produce circulating factors, there is no doubt that other signaling molecules that we have not measured, and probably yet others that we do not even know about at present, are also changed by these alterations in tissue size (indicated by …. OTHERS, at the relevant points of Figure 14). These circulating factors are probably integral components of the energy balance sensing and regulation system in the hypothalamic nuclei and the body temperature regulation system located in the pre-optic area of the hypothalamus (red arrows) [27, 66, 67]. The consequence of the declining hormone levels is that the body temperature regulation system located in the pre-optic area (POA) reduces the mean daily body temperature in relation to the hormone levels. This may be a direct effect on the POA since there are abundant leptin receptors on neurons in the POA [68], and receptors for other signaling compounds from the periphery such as TNF-α and IL-6 [27], or an indirect effect mediated from the arcuate, ventromedial and dorsomedial nuclei of the energy homeostasis system [69]. Since there are also projections from the POA to the dorsomedial nuclei [70] a reciprocal interaction seems likely. It has also been suggested that key components of the temperature regulation system in the POA, in the context of CR effects, may include sensitization of adenosine 1A receptors [71] and the microglia [51].

At critically low levels of BM, indicating extremely low body energy stores, the temperature regulation system also initiates the use of torpor (Figures 5 and 9). Low leptin levels appeared to be a key signaling factor initiating this response. Leptin has previously been strongly implicated in regulation of torpor [72]. *Ob/Ob* mice lacking functional leptin enter torpor readily when food deprived, despite having large fat stores [73, 74]. This torpor response can be completely abolished by treating with exogenous leptin [75]. However, leptin alone appears insufficient to regulate torpor levels since it does not abolish torpor in A-ZIP/F-1 mice which lack white adipose tissue [75]. Testosterone and prolactin [76], norepinephrine [77], sirtuins and fibroblast growth factor 21 [78] have all been implicated as additional important signaling molecules in torpor initiation. Since we did not measure these factors in the present study, their role in CR induced torpor in our mice is uncertain. However, as structural tissue levels were the primary factor governing the occurrence of torpor in our mice, uncharacterized circulating factors related to these tissues will likely prove an important mechanism initiating torpor in CR. Body temperature during torpor was lower in mice under 40CR compared to mice under 30CR and became lower as the duration of restriction increased. This suggested the depth of torpor was also under regulation by unknown or uncharacterized factors. Previous work has implicated ghrelin and the neuropeptide Y (NPY) signaling system in the arcuate nucleus as playing a key role in regulating torpor depth [79].

Both lowered body temperature and torpor contribute to reduced energy expenditure, which ultimately balances the reduced intake. At this point the mobilization of the tissue ceases, but the sizes of the reduced tissues mean that all the signaling molecules produced by these tissues remain affected and hence the reduced body temperature and use of torpor are sustained. The main shortfall of this model is to understand the changes in body temperature under CR that do not closely match the alterations in body composition and resultant signals. Is this a consequence of the complex interplay of different signaling molecules from the body tissue compartments as the animals lose BM? Alternatively there may be additional factors impinging temperature regulation independent of the body composition for example ambient temperature (indicated in Figure 14 as ‘other factors?’).

This model is consistent with the observations of both the body composition [43], hormonal changes [44] and body temperature responses (this paper) of the animals that were placed on matched levels of PR. In these PR animals we observed no reductions in body temperature under restriction (Figures 2b and 12). Following the model in Figure 14 this absence of an effect under PR would be because there was no energy imbalance when fed these diets, hence there was no mobilization of the body tissue (confirmed in [43]) to precipitate changed levels of the circulating hormones (confirmed in [44]) that would then stimulate the reduced body temperature, and ultimately use of torpor (observed here).

## MATERIALS AND METHODS

### Overall design and rationale

The overall project rationale has been presented elsewhere [43]. In the current paper we characterized the body temperature response to CR and PR in C57BL/6 male mice, a strain known to have a positive lifespan response under CR [10]. Mice were introduced to CR or PR at 20 weeks of age, approximately equivalent to early human adulthood. A linear relationship between the extent of CR and the magnitude of the lifespan effect has been indicated, up to at least a restriction of 65% which led to a 60% increase in lifespan [41, 42, 80]. We therefore exposed mice to 5 different levels of CR: 0, 10, 20, 30 and 40% lower calories than their own individual intakes measured over a baseline period of 14 days prior to introducing the restricted diets (*n* = 8 or 9 per group). Mice on restriction were individually housed and fed daily at lights out (1830). We used two different control groups exposed to 0% CR. For the first group (24AL) we allowed them 24 hr access to food without restriction. For the second group (12AL) we allowed them unrestricted access to food for the 12 hr of darkness but the food was then removed at lights on (0630), replacing it 12 hr later at lights off when the CR animals were also fed. Mice fed completely *ad libitum* (AL) tend to overeat which can lead to obesity & related morbidities. Consequently, the reliability of using 24AL groups as controls in CR studies is questionable [2, 81]. This 12 hour feeding regime also ensures all mice have been fasted for a minimum of 7.5 hr when culled from 1400 and eliminates skewed results in for example, hormone levels from control mice which may have fed prior to cull [43].

We fed mice every day since previous work has indicated frequency of feeding may affect their body temperature responses [26].

All animals were fed a high carbohydrate open source diet (D12450B: Research diets, NJ, USA) which contains 20% protein, 70% carbohydrate and 10% fat (by energy). For the animals on PR we used the same diet containing 20% protein as the control group. We fed additional groups diets designed to match the baseline diet but with reduced protein contents equal to 16, 14 and 12% protein (made up by increased carbohydrate) (D13020201, D13020202 and D13020203 respectively, Research Diets, NJ, USA). These animals were prevented from overeating to compensate for the reduced protein and were fed a fixed weight of food equivalent to their own individual baseline intake on the 20% protein diet. Hence their energy intakes were the same as during the baseline period but their protein intakes were restricted by 20, 30 and 40%, to match the protein levels consumed by the 20, 30 and 40% CR groups. The diets used, control (D12450B) and protein restricted, were specifically designed to be isocaloric with a calorie content of 3.8kcal/g. To match the CR protocol these animals were also only fed in darkness. For both studies the period of restriction was set at 3 months. The overall aim of the study was to collect extensive phenotype data across the 7–9 animals in each group. These data included transcriptomic, proteomic and metabolomic profiles in multiple tissues, physiological, endocrinological and behavioral responses, as well as morphological changes. The focus of the current paper is the effects of CR and PR on daily average body temperature and the occurrence of torpor.

### Animals

All procedures were reviewed and approved by University of Aberdeen ethical approval committee and carried out under a Home Office issued license compliant with the Animals (Scientific Procedures) Act 1986. C57BL/6 mice were purchased from Charles River (Ormiston, UK). Free access to water was provided. Mice were allocated into experimental groups matched for body mass (BM). Ambient temperature was maintained at 21 ± 1^°^C.

### Body temperature

Core body temperature (and physical activity) were measured using the VitalView™ telemetry and data acquisition system (MiniMitter, OR, USA) as used previously [82]. The transmitters, implanted intraperitoneally, are unrestrictive allowing free movement of the animals. Minute by minute recordings are transmitted via an ER-4000 receiving platform and VitalView™ software was used to acquire data (MiniMitter, OR, USA). All surgeries were performed at 12 weeks of age allowing adequate recovery time prior to experimentation. Due to a malfunction of 2 transmitters *n* = 8 for all groups bar 30CR where *n* = 6. Animals were undisturbed bar feeding, weighing and routine checks. For a full description of the system refer to [83].

### Statistical analysis

Statistical analyses were performed using the PASW Statistics package 18, Minitab version 16 and the R statistical environment. Visual inspection of the responses in body temperature over time suggested that there was a dynamic phase followed by a more stable phase. We analyzed these sections separately. Within each section we explored the effects of level of CR using general linear models (GLM). We included the day of restriction as a covariate and individual nested within restriction group as a random factor to account for the fact we had repeated measurements (RM) of the individuals. When the treatment group was significant we located the pairwise differences using post hoc Tukey tests. During the first phase when the body temperature was changing dynamically with day, we fitted quadratic equations to the data (day and day^2^ as predictors) and summarized salient features of the changes using parameters derived from these fitted equations—specifically the time to reach the nadir in temperature and the initial rate of temperature change at the onset of restriction. To explore the relationships between body temperature over the last 20 days of the experiment and morphological and circulating hormonal factors at the end of the experiment we used least squares single and multiple regression analysis, eliminating non-significant terms using a backward elimination stepwise procedure. Linear regression analyses were verified by exploration of the diagnostic plots of residuals against fitted values. We explored temporal patterning in the body temperature responses using autocorrelation and partial autocorrelation analysis. In all tests *p* < 0.05 was regarded as significant.

## SUPPLEMENTARY FIGURE



## References

[1] Weindruch R, Walford RL (1982). Dietary restriction in mice beginning at 1 year of age: Effect on life-span and spontaneous cancer incidence. Science.

[2] Speakman JR, Mitchell SE (2011). Caloric restriction. Mol Aspects Med.

[3] Osborne TB, Mendel LB, Ferry EL (1917). The effect of retardation of growth upon the breeding period and duration of life in rats. Science.

[4] Weindruch R, Walford RL, Thomas C.C. (1988). The retardation of aging and disease by dietary restriction.

[5] Colman RJ, Anderson RM, Johnson SC, Kastman EK, Kosmatka KJ, Beasley TM, Allison DB, Cruzen C, Simmons HA, Kemnitz JW, Weindruch R (2009). Caloric restriction delays disease onset and mortality in rhesus monkeys. Science.

[6] Colman RJ, Beasley TM, Kemnitz JW, Johnson SC, Weindruch R, Anderson RM (2014). Caloric restriction reduces age-related and all-cause mortality in rhesus monkeys. Nature Communications.

[7] Cooper TM, Mockett RJ, Sohal BH, Sohal RS, Orr WC (2004). Effect of caloric restriction on life span of the housefly, musca domestica. FASEB J.

[8] Mattison JA, Roth GS, Beasley TM, Tilmont EM, Handy AM, Herbert RL, Longo DL, Allison DB, Young JE, Bryant M, Barnard D, Ward WF, Qi W (2012). Impact of caloric restriction on health and survival in rhesus monkeys from the NIA study. Nature.

[9] Austad SN (2012). Ageing: Mixed results for dieting monkeys. Nature.

[10] Turturro A, Witt WW, Lewis S, Hass BS, Lipman RD, Hart RW (1999). Growth curves and survival characteristics of the animals used in the biomarkers of aging program. The Journals of Gerontology Series A: Biological Sciences and Medical Sciences.

[11] Forster M, Morris P, Sohal R (2003). Genotype of age influence the effect of caloric intake on mortality in mice. FASEB J.

[12] Sohal RS, Ferguson M, Sohal BH, Forster MJ (2009). Life span extension in mice by food restriction depends on an energy imbalance. J Nutr.

[13] Liao C, Rikke BA, Johnson TE, Diaz V, Nelson JF (2010). Genetic variation in the murine lifespan response to dietary restriction: From life extension to life shortening. Aging Cell.

[14] Hempenstall S, Picchio L, Mitchell SE, Speakman JR, Selman C (2010). The impact of acute caloric restriction on the metabolic phenotype in male C57BL/6 and DBA/2 mice. Mech Ageing Dev.

[15] Mulvey L, Sinclair A, Selman C (2014). Lifespan modulation in mice and the confounding effects of genetic background. Journal of Genetics and Genomics.

[16] Millet L, Vidal H, Andreelli F, Larrouy D, Riou JP, Ricquier D, Laville M, Langin D (1997). Increased uncoupling protein-2 and -3 mRNA expression during fasting in obese and lean humans. J Clin Invest.

[17] Picard F, Guarente L (2005). Molecular links between aging and adipose tissue. Int J Obes.

[18] Bordone L, Guarente L (2005). Calorie restriction, SIRT1 and metabolism: Understanding longevity. Nat Rev Mol Cell Biol.

[19] Colom B, Oliver J, Roca P, Garcia-Palmer FJ (2007). Caloric restriction and gender modulate cardiac muscle mitochondrial H2O2 production and oxidative damage. Cardiovasc Res.

[20] Masoro EJ (2009). Caloric restriction-induced life extension of rats and mice: A critique of proposed mechanisms. Biochim Biophys Acta.

[21] Barzilai N, Bartke A (2009). Biological approaches to mechanistically understand the healthy life span extension achieved by calorie restriction and modulation of hormones. The Journals of Gerontology Series A: Biological Sciences and Medical Sciences.

[22] Weindruch RH, Kristie JA, Cheney KE, Walford RL (1979). Influence of controlled dietary restriction on immunologic function and aging. Fed Proc.

[23] Duffy PH, Feuers RJ, Leakey JA, Nakamura KD, Turturro A, Hart RW (1989). Effect of chronic caloric restriction on physiological variables related to energy metabolism in the male fischer 344 rat. Mech Ageing Dev.

[24] Duffy PH, Feuers R, Nakamura KD, Leakey J, Hart RW (1990). Effect of chronic caloric restriction on the synchronization of various physiological measures in old female fischer 344 rats. Chronobiol Int.

[25] Duffy PH, Leakey JEA, Pipkin JL, Turturro A, Hart RW (1997). The physiologic, neurologic, and behavioral effects of caloric restriction related to aging, disease, and environmental factors. Environ Res.

[26] Rikke BA, Yerg JE, Battaglia ME, Nagy TR, Allison DB, Johnson TE (2003). Strain variation in the response of body temperature to dietary restriction. Mech Ageing Dev.

[27] Tabarean I, Morrison B, Marcondes MC, Bartfai T, Conti B (2010). Hypothalamic and dietary control of temperature-mediated longevity. Ageing Research Reviews.

[28] Bartfai T, Conti B (2012). Molecules affecting hypothalamic control of core body temperature in response to calorie intake. Frontiers in Genetics.

[29] Lane MA, Baer DJ, Rumpler WV, Weindruch R, Ingram DK, Tilmont EM, Cutler RG, Roth GS (1996). Calorie restriction lowers body temperature in rhesus monkeys, consistent with a postulated anti-aging mechanism in rodents. Proceedings of the National Academy of Sciences.

[30] Roth GS, Lane MA, Ingram DK, Mattison JA, Elahi D, Tobin JD, Muller D, Metter EJ (2002). Biomarkers of caloric restriction may predict longevity in humans. Science.

[31] Mattison JA, Lane MA, Roth GS, Ingram DK (2003). Calorie restriction in rhesus monkeys. Exp Gerontol.

[32] Soare A, Cangemi R, Omodei D, Holloszy JO, Fontana L (2011). Long-term calorie restriction, but not endurance exercise, lowers core body temperature in humans. Aging (Albany NY).

[33] Heilbronn LK, de Jonge L, Frisard MI, DeLany JP, Larson-Meyer DE, Rood J, Nguyen T, Martin CK, Volaufova J, Most MM, Greenway FL, Smith SR, Deutsch WA (2006). Effect of 6-month calorie restriction on biomarkers of longevity, metabolic adaptation, and oxidative stress in overweight individuals: A randomized controlled trial. JAMA.

[34] Redman LM, Martin CK, Williamson DA, Ravussin E (2008). Effect of caloric restriction in non-obese humans on physiological, psychological and behavioral outcomes. Physiol Behav.

[35] Conti B, Sanchez-Alavez M, Winsky-Sommerer R, Morale MC, Lucero J, Brownell S, Fabre V, Huitron-Resendiz S, Henriksen S, Zorrilla EP, de Lecea L, Bartfai T (2006). Transgenic mice with a reduced core body temperature have an increased life span. Science.

[36] Koizumi A, Wada Y, Tuskada M, Kayo T, Naruse M, Horiuchi K, Mogi T, Yoshioka M, Sasaki M, Miyamaura Y (1996). A tumor preventive effect of dietary restriction is antagonized by a high housing temperature through deprivation of torpor. Mech Ageing Dev.

[37] Hunter W, Croson W, Bartke A, Gentry M, Meliska C (1999). Low body temperature in long-lived ames dwarf mice at rest and during stress. Physiol Behav.

[38] Walford RL, Liu RK (1965). Husbandry, life span, and growth rate of the annual fish, cynolebias adloffi E. ahl. Exp Gerontol.

[39] Liu RK, Walford RL (1966). Increased growth and life-span with lowered ambient temperature in the annual fish, cynolebias adloffi. Nature.

[40] Walford RL (1983). Maximum life span.

[41] Merry B (2002). Molecular mechanisms linking calorie restriction and longevity. Int J Biochem Cell Biol.

[42] Speakman JR, Hambly C (2007). Starving for life: What animal studies can and cannot tell us about the use of caloric restriction to prolong human lifespan. J Nutr.

[43] Mitchell SE, Tang ZH, Kerbois C, Delville C, Konstantopedos P, Bruel A, Derous D, Green C, Aspden RM, Goodyear SR, Chen L, Han JJD, Wang Y (2015). The effects of graded levels of calorie restriction: I. impact of short term calorie and protein restriction on body composition in the C57BL/6 mouse. Oncotarget.

[44] Mitchell SE, Delville C, Konstantopedos P, Hurst J, Derous D, Green C, Chen L, Han JJD, Wang Y, Promislow DEL, Lusseau D, Douglas A, Speakman JR (2015). The effects of graded levels of calorie restriction: II. impact of short term calorie and protein restriction on oxidative stress, circulating adipokine levels and glucose homeostasis in the C57BL/6 mouse. Oncotarget.

[45] Golightly A, Boys RJ, Cameron KM, Zglinicki Tv (2012). The effect of late onset, short-term caloric restriction on the core temperature and physical activity in mice. Journal of the Royal Statistical Society: Series C (Applied Statistics).

[46] Lusseau D, Mitchell SE, Barros C, Derous D, Green C, Chen L, Han JJD, Wang Y, Promislow DEL, Douglas A, Speakman JR (2015). The effect of graded levels of calorie restriction IV: Non linear change in behavioural phenotype of male C57BL/6 mice in response to short-term graded calorie restriction. Scientific Reports.

[47] Simpson SJ, Raubenheimer D (2009). Macronutrient balance and lifespan. Aging (Albany NY).

[48] Hudson JW, Scott IM (1979). Daily torpor in the laboratory mouse, mus musculus var. albino. Physiol Zool.

[49] Rikke BA, Johnson TE (2004). Lower body temperature as a potential mechanism of life extension in homeotherms. Exp Gerontol.

[50] MacDonald L, Hazi A, Paolini AG, Kent S (2014). Calorie restriction dose-dependently abates lipopolysaccharide-induced fever, sickness behavior, and circulating interleukin-6 while increasing corticosterone. Brain Behav Immun.

[51] Radler ME, Hale MW, Kent S (2014). Calorie restriction attenuates lipopolysaccharide (LPS)-induced microglial activation in discrete regions of the hypothalamus and the subfornical organ. Brain Behav Immun.

[52] Conti B (2008). Considerations on temperature, longevity and aging. Cell Mol Life Sci.

[53] Schmid J, Speakman J (2000). Daily energy expenditure of the grey mouse lemur (microcebus murinus): A small primate that uses torpor. Journal of Comparative Physiology B.

[54] Canale CI, Perret M, Henry P (2012). Torpor use during gestation and lactation in a primate. Naturwissenschaften.

[55] Cameron KM, Golightly A, Miwa S, Speakman J, Boys R, von Zglinicki T (2011). Gross energy metabolism in mice under late onset, short term caloric restriction. Mech Ageing Dev.

[56] Guarente L, Picard F (2005). Calorie restriction: The SIR2 connection. Cell.

[57] Libert S, Guarente L (2013). Metabolic and neuropsychiatric effects of calorie restriction and sirtuins. Annu Rev Physiol.

[58] Elia M (1992). Organ and tissue contribution to metabolic rate. Energy Metabolism: Tissue Determinants and Cellular Corollaries.

[59] Johnstone AM, Murison SD, Duncan JS, Rance KA, Speakman JR (2005). Factors influencing variation in basal metabolic rate include fat-free mass, fat mass, age, and circulating thyroxine but not sex, circulating leptin, or triiodothyronine. The American Journal of Clinical Nutrition.

[60] Greenberg J, Boozer C (2000). Metabolic mass, metabolic rate, caloric restriction, and aging in male fischer 344 rats. Mech Ageing Dev.

[61] Kaiyala KJ, Morton GJ, Leroux BG, Ogimoto K, Wisse B, Schwartz MW (2010). Identification of body fat mass as a major determinant of metabolic rate in mice. Diabetes.

[62] Chen H, Luo L, Liu J, Brown T, Zirkin BR (2005). Aging and caloric restriction: Effects on leydig cell steroidogenesis. Exp Gerontol.

[63] Harper JM, Leathers CW, Austad SN (2006). Does caloric restriction extend life in wild mice?. Aging Cell.

[64] Rocha JS, Bonkowski MS, Franca LR, Bartke A (2007). Mild calorie restriction does not affect testosterone levels and testicular gene expression in mutant mice. Exp Biol Med (Maywood).

[65] Sitzmann BD, Leone EH, Mattison JA, Ingram DK, Roth GS, Urbanski HF, Zelinski MB, Ottinger MA (2010). Effects of moderate calorie restriction on testosterone production and semen characteristics in young rhesus macaques (macaca mulatta). Biol Reprod.

[66] Hammel HT, Hardy JD, Fusco MM (1960). Thermoregulatory responses to hypothalamic cooling in unanesthetized dogs. Am J Physiol.

[67] Hammel HT, Jackson DC, Stolwijk JA, Hardy JD, Stromme SB (1963). Temperature regulation by hypothalamic proportional control with an adjustable set point. J Appl Physiol.

[68] Zhang Y, Kerman IA, Laque A, Nguyen P, Faouzi M, Louis GW, Jones JC, Rhodes C, Munzberg H (2011). Leptin-receptor-expressing neurons in the dorsomedial hypothalamus and median preoptic area regulate sympathetic brown adipose tissue circuits. J Neurosci.

[69] Dacks PA, Moreno CL, Kim ES, Marcellino BK, Mobbs CV (2013). Role of the hypothalamus in mediating protective effects of dietary restriction during aging. Front Neuroendocrinol.

[70] Nakamura K, Morrison SF (2007). Central efferent pathways mediating skin cooling-evoked sympathetic thermogenesis in brown adipose tissue. Am J Physiol Regul Integr Comp Physiol.

[71] Jinka TR, Carlson ZA, Moore JT, Drew KL (2010). Altered thermoregulation via sensitization of A1 adenosine receptors in dietary-restricted rats. Psychopharmacology.

[72] Geiser F, Kortner G, Schmidt I (1998). Leptin increases energy expenditure of a marsupial by inhibition of daily torpor. Am J Physiol.

[73] Trayhurn P, James WPT (1978). Thermoregulation and non-shivering thermogenesis in the genetically obese (ob/ob) mouse. Pflügers Archiv European Journal of Physiology.

[74] Ahima RS, Prabakaran D, Mantzoros C, Qu D, Lowell B, Maratos-Flier E, Flier JS (1996). Role of leptin in the neuroendocrine response to fasting. Nature.

[75] Gavrilova O, Leon LR, Marcus-Samuels B, Mason MM, Castle AL, Refetoff S, Vinson C, Reitman ML (1999). Torpor in mice is induced by both leptin-dependent and -independent mechanisms. Proceedings of the National Academy of Sciences of the United States of America.

[76] Ruby NF, Nelson RJ, Licht P, Zucker I (1993). Prolactin and testosterone inhibit torpor in siberian hamsters. Am J Physiol.

[77] Swoap SJ, Weinshenker D (2008). Norepinephrine controls both torpor initiation and emergence via distinct mechanisms in the mouse. PLoS One.

[78] Melvin RG, Andrews MT (2009). Torpor induction in mammals: Recent discoveries fueling new ideas. Trends in Endocrinology & Metabolism.

[79] Gluck EF, Stephens N, Swoap SJ (2006). Peripheral ghrelin deepens torpor bouts in mice through the arcuate nucleus neuropeptide Y signaling pathway. Am J Physiol Regul Integr Comp Physiol.

[80] Weindruch R, Walford RL, Fligiel S, Guthrie D (1986). The retardation of aging in mice by dietary restriction: Longevity, cancer, immunity and lifetime energy intake. J Nutr.

[81] Sohal RS, Forster MJ (2014). Caloric restriction and the aging process: A critique. Free Radical Biology and Medicine.

[82] Gamo Y, Troup C, Mitchell SE, Hambly C, Vaanholt LM, Speakman JR (2013). Limits to sustained energy intake. XX. body temperatures and physical activity of female mice during lactation. J Exp Biol.

[83] Harkin A, O'Donnell JM, Kelly JP (2002). A study of VitalView™ for behavioural and physiological monitoring in laboratory rats. Physiol Behav.

