# Supplementary material for: The effects of graded levels of calorie restriction: III. Impact of short term calorie and protein restriction on mean daily body temperature and torpor use in the C57BL/6 mouse
Source: Oncotarget. 2015 Jul 22;6(21):18314–37. doi: 10.18632/oncotarget.4506 (PMC4621893; doi:10.18632/oncotarget.4506)
Supplement: Supplementary file 1 [file oncotarget-06-18314-s001.pdf]

SUPPLEMENTARY FIGURE

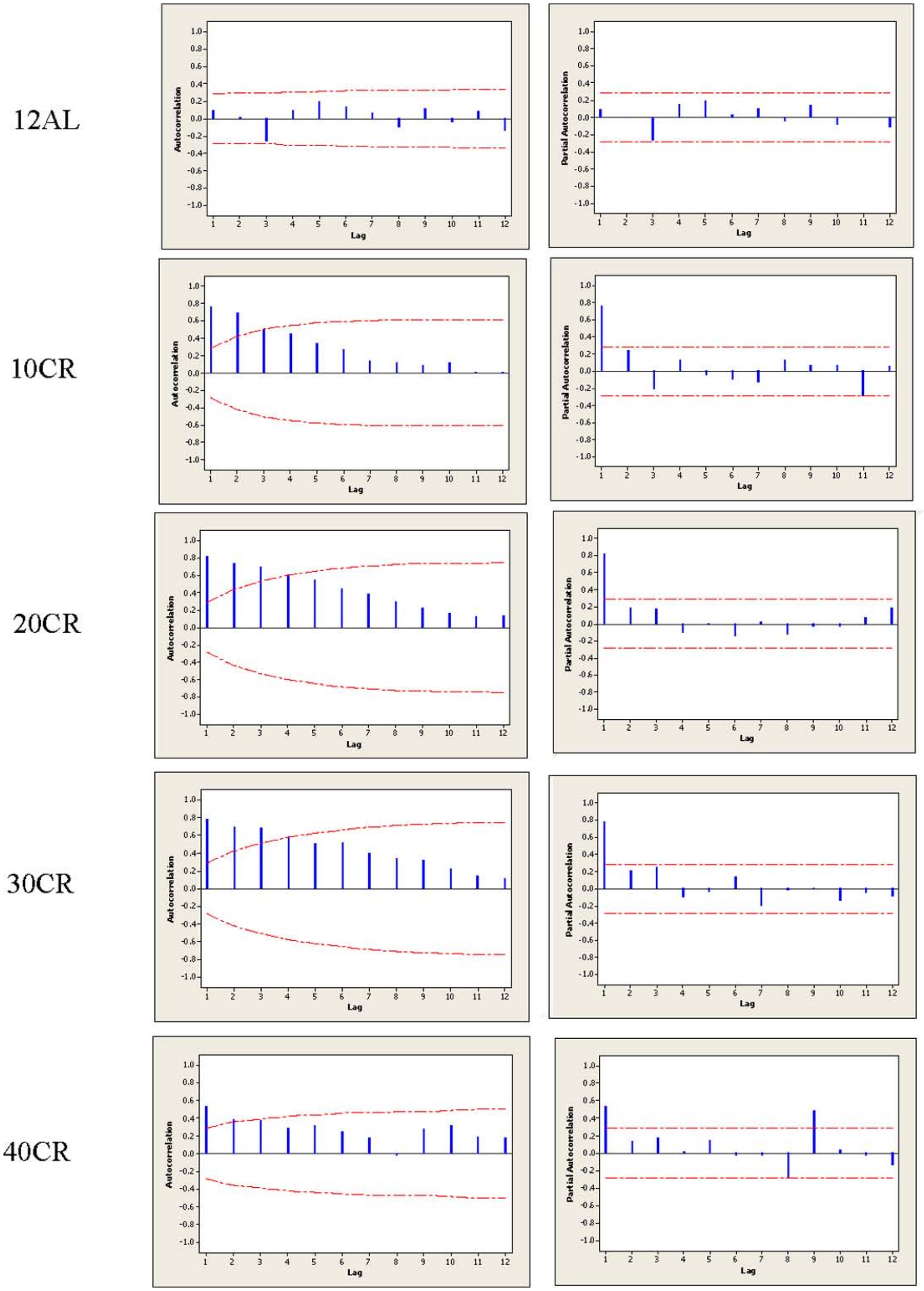

Supplementary Figure S1: Autocorrelation and partial autocorrelation plots for the time series of body temperature measurements following the initial dynamic phase of change. In each plot the correlation at various lag times is shown as a blue bar and the relevant significant levels are shown by dotted red lines. The plots indicated significant autocorrelation between days only with a lag of 1 day.
